# Cotard’s Delusion and Its Relation With Different Psychiatric Diagnoses in a Tertiary Care Hospital

**DOI:** 10.7759/cureus.39477

**Published:** 2023-05-25

**Authors:** Sabita Dihingia, Dhrubajyoti Bhuyan, Mridusikha Bora, Nikhita Das

**Affiliations:** 1 Department of Psychiatry, Nalbari Medical College & Hospital, Nalbari, IND; 2 Department of Psychiatry, Assam Medical College & Hospital, Dibrugarh, IND

**Keywords:** nihilistic delusion, negation, suicidal thoughts, psychotic disorder, depression, nihilism

## Abstract

Background

Cotard's delusion/Cotard's syndrome is a series of delusions ranging from a false, fixed, unshakeable belief that one has lost their soul, blood, organs, and body parts to the belief that one is dead. The syndrome was initially thought to be associated with only mood disorders but later was found in other psychiatric illnesses as well.

Aim

The study aimed to find an association between Cotard's delusion and the psychopathology of different psychiatric diagnoses.

Method

The clinical study comprised seven patients presenting with symptoms of Cotard syndrome with different presentations, diagnoses, and onset and meeting inclusion criteria. The study was carried out in the Department of Psychiatry, Assam Medical College and Hospital. The patients were hospitalized and, after a detailed history, mental status examination, and laboratory investigations, were treated with pharmacological and non-pharmacological methods. A descriptive statistical analysis was done.

Results

Denial of the existence of body organs was the most similar complaint encountered in the cases. The duration of illness onset ranged from weeks to months. The symptoms were found to be present in different psychiatric illnesses like schizophrenia, delusional disorder, depression, and intellectual disability. The patient had responded well to pharmacological agents with the exception of three patients who were treated with electroconvulsive therapy.

Conclusion

The study highlights the different subtypes of Cotard’s syndrome and its associated symptoms, which provides a better understanding of the condition. The case series presents a finding of a higher proportion of male patients and adolescent cases than in previous reports. The study also provides valuable insights into its heterogeneity in the diagnosis and treatment of Cotard’s syndrome, which may help in the early recognition and management of this rare condition.

## Introduction

As described by Jules Cotard, Cotard's delusion/Cotard's syndrome is a series of delusions varying from false, fixed, unshakeable beliefs that there is no existence of blood, organs, or body parts or that one is dead [1]. He initially characterized it as the delirium of negation (le délire des négations) and believed that he has discovered a new entity of depression, which was accompanied by anxiety, hypochondriacal ideas, absence of organs or soul, immortality as well as self-harm behavior [2]. Later, others found that the syndrome can be associated with other psychological and psychiatric disorders. Regis termed this syndrome as délire de cotard (Cotard’s delirum) [3]. The syndrome has been mostly reported in patients with depression with suicidal thoughts, schizophrenia, or bipolar manic-depressive psychosis [4], and in some patients diagnosed with catatonia [5-7]. A few case reports have also highlighted Cotard's syndrome in patients presenting with self-starvation and nutritional deficiencies [8]. Studies have reported that the co-occurrence of schizophrenia and Cotard's syndrome is rare [9]. In this clinical study, we report seven series of cases of Cotard's syndrome with different presentations, diagnoses, onset, and treatment received with the aim to find an association between Cotard's delusion and the psychopathology of different diagnoses.

## Materials and methods

Seven patients meeting the presentation of Cotard's syndrome with different diagnoses according to the International Classification of Disease, 10th Edition, were selected for this clinical study. The study was carried out in the Department of Psychiatry, Assam Medical College and Hospital. Permission for ethical approval was taken from the Institutional Ethics Committee (H), bearing number 2023/AMC/EC/2404. Two senior psychiatrists were involved in establishing the diagnosis. The demographic and clinical data were obtained from the patients during the interview. Patients who had given their informed consent and assent were included in the study while patients who had serious deteriorating illnesses were excluded from the study. The included patients were assessed every week by at least one senior psychiatrist to see the progression of the symptoms. A detailed workup involving history, serial mental status examinations, and laboratory investigations was carried out and treated with pharmacological and non-pharmacological methods.

## Results

Among all the patients included in the study, four of the seven patients were males, with a mean age of 40.7 and a standard deviation of 20.42. The duration of symptoms ranged from 15 days to 6 months. Denial of the existence of body organs was the most common complaint, which was experienced by five patients. One patient among the seven had a history of similar symptoms. Five patients had met the diagnosis of a psychotic disorder whereas two patients were diagnosed with a mood disorder. Two patients had suicidal ideation and had attempted to take their life. All the patients had received inpatient treatment and compliance was satisfactory in every case. The patients or family members did not report any side effects either from the pharmacological or somatic treatment.

Case series

Patient 1

A 70-year-old male presented with complaints of withdrawn behavior, disturbed sleep, fearfulness, and suspiciousness for two months. As his symptoms increased, he developed delusions of persecution and nihilism. The content of nihilistic delusion was that the world was coming to an end, and human extinction is inevitable. He could vibe that an apocalypse is taking place and therefore his brain has stopped functioning. On mental status examination, he had findings of irritable affect, increased psychomotor activity, and persecutory and nihilistic delusions. He was initially treated on injectable antipsychotics and started on electroconvulsive therapy, as his suicidal thoughts were becoming worse. He received seven electroconvulsive therapy treatments. The patient was also concomitantly started on tab aripiprazole 5 mg, which was later increased to 10 mg. In six weeks, the patient showed gradual improvement in his suicidal thoughts and nihilistic ideas. He was discharged after eight weeks.

Patient 2

A 45-year-old female presented with complaints of withdrawn behavior, the belief that her head has rotated, her stomach was twisted and has vanished, decreased sleep, and belief about having a matchstick inside her ear for six months. On mental status examination, she had constricted affect with nihilistic delusions, somatic delusions, and a sense of helplessness and hopelessness. She was diagnosed with persistent delusional disorders according to the International Classification of Disease, 10th revision [10], and delusion of guilt. She scored 26 on applying Beck’s Depression Inventory indicating moderate depression. She was started on tablet Pimozide 8 mg in two divided doses and a combination of tablet escitalopram 5 mg and clonazepam 0.5 mg at bedtime. After two months, the Hamilton Depression Rating scale was applied to check the progression of her disease with a score of 10 indicating mild depression and a delusion of nihilism being shakable. After another month, she had significant improvement in her content of thought and was continued on the same medications.

Patient 3

A 65-year-old male presented to the Department of Psychiatry with complaints of low mood, lack of interest in previously enjoyable activities, withdrawn behavior, disturbed sleep, and feeling that his body is rotten for 45 days. On detailed evaluation and mental status examination, the patient was found to be depressed and had secondary delusions that were of a nihilistic type. He was diagnosed with a severe depressive episode with psychotic symptoms (F32.3) according to ICD 10 and was immediately hospitalized. He also had suicidal thoughts and also had tried once to end his life. He was given seven electroconvulsive therapy treatments and simultaneously started on tab vortioxetine 10 mg in two divided doses and tablet aripiprazole 10 mg at bedtime. He was discharged after eight weeks, as he showed significant improvement in his symptoms.

Patient 4

A 35-year-old female had a previous similar episode of mental illness 20 years back. She presented with disturbed sleep, withdrawn behavior, seeing things not seen by others, irrelevant talk, and feeling that the earth is going to end in three years for the last 15 days. She further elaborated that she could see the rivers dry up in her vision when she was not sleeping, and mountains collapsing. She believed that everyone around her was not there and her soul has left her body. On mental status examination, she had nihilistic delusions and visual hallucinations with poor insight. She fulfilled the criteria for acute schizophrenia-like psychotic disorder and was started on tablet Olanzapine 10 mg for two weeks and then was titrated to Olanzapine 20 mg at bedtime. After four weeks, she was assessed again, where her nihilistic beliefs and hallucination subsided.

Patient 5

A 30-year-old married man, hailing from a village, presented with three months of history of suspiciousness, withdrawn behavior, disturbed sleep, and alleged history of one attempt at taking his own life at his residence. His developmental milestone was found to be delayed as compared to the children of the neighborhood. His intelligence quotient came out to be 44 after applying standardized IQ tests. On further asking, the patient replied that both his kidneys were absent and had been taken by someone and since he has no kidneys, he wished to die. He was diagnosed as a case of schizophrenia (F20) with an intellectual disability. He was admitted to the inpatient Psychiatry Department. He was started on tablet risperidone 6 mg in two divided doses and lorazepam 2 mg at bedtime for two months. He came for regular follow-ups and maintained improvement on the same treatment.

Patient 6

A 24-year-old male presented with difficulty in breathing and complained that he is going to die. He was admitted and on detailed evaluation, he was thought to be a case of dissociative disorder. To decrease his hyperventilation, benzodiazepine was started. After his symptoms decreased, he still firmly believed that he would die, as his internal organs are rotting. On further evaluation, findings of irritable affect, persecutory delusion, nihilistic delusions, and delusion of reference in content; third-person auditory hallucinations were present with poor insight. He was diagnosed with paranoid schizophrenia (F20.0). He was admitted and received seven electroconvulsive therapies. Simultaneously, he was also started on tab risperidone 6 mg in two divided doses, which was later increased to 8 mg. He was discharged after two months when his symptoms improved.

Patient 7

A 16-year-old female, resident of Lekhapani, Tinsukia, and student of class 10 was brought to the Psychiatry Department of Assam Medical College and Hospital on August 27, 2019, with chief complaints of low mood, easy fatiguability, irrelevant talk, fearfulness, decreased sleep, decreased appetite, one attempt of self-harming behavior with acute onset and progressive course with mental status examination findings such as personal hygiene and grooming not maintained with blunt affect, restricted range toward the sad side, poverty of speech and ideas of guilt, denial of the existence of self and organs, and delusion of reference in content of thought. She had no insight into her illness and was diagnosed as a case of severe depressive episode with psychotic symptoms. She was started on tablet Olanzapine 10 mg and tablet mirtazapine 10 mg. After receiving eight ECTS, her symptoms improved and she was discharged. 

The cases have been summarized in Table 1 and Table 2.

**Table 1:** Description of presenting complaints

Patient	Presenting Complaints
Patient 1	Withdrawn behavior, disturbed sleep, fearfulness, and suspiciousness
Patient 2	Withdrawn behavior, the belief that the head has rotated, the stomach twisted and vanished, decreased sleep
Patient 3	Low mood, lack of interest in previously enjoyable activities, withdrawn behavior, disturbed sleep, and feeling that body has rotten
Patient 4	Disturbed sleep, withdrawn behavior, seeing things not seen by others, irrelevant talk, and feeling that the earth will end
Patient 5	Suspiciousness, withdrawn behavior, disturbed sleep, and suicidal attempt
Patient 6	Difficulty in breathing and death wish
Patient 7	Low mood, easy fatiguability, irrelevant talk, fearfulness, decreased sleep, decreased appetite, and suicidal attempt

**Table 2:** Case summary of patients

Patient	Age (in years)	Gender	Duration	Delusional content	Diagnosis	Treatment
Patient 1	70	Male	2 months	Denial of the existence of organs: liver and brain Denial of the existence of the world	Schizophrenia (ICD 10 - F20)	Aripiprazole = 10mg, Electroconvulsive Therapy
Patient 2	45	Female	6 months	Denial of the existence of organ: stomach	Persistent Delusional Disorder (ICD 10 – F25)	Pimozide = 16mg, Escitalopram = 5mg, Clonazepam = 0.25mg
Patient 3	65	Male	1.6 months	Denial of the existence of organs inside the abdomen	Severe Depressive Disorder (ICD 10 -F32.2)	Vortioxetine = 20mg, Aripiprazole = 10mg, Electroconvulsive Therapy
Patient 4	35	Female	15 days	Denial of bodily existence and soul Denial of existence in the world	Acute and Transient Psychotic Disorder (ICD 10 -F23)	Olanzapine = 20mg
Patient 5	30	Male	3 months	Denial of the existence of organs: kidneys	Schizophrenia + Moderate mental retardation	Risperidone = 6mg, Trihexyphenidyl = 2mg
Patient 6	24	Male	30 days	Denial of the existence of organs inside the abdomen	Schizophrenia (ICD 10 – F20)	Risperidone = 6mg, Trihexyphenidyl = 2mg
Patient 7	16	Female	3 months	Denial of the existence of self, Denial of the existence of organs	Severe Depressive Disorder with psychotic symptoms (ICD 10 - F32.3)	Olanzapine = 10mg, Mirtazapine = 10mg, Electroconvulsive Therapy

## Discussion

Cotard’s syndrome has been found to be associated with different psychiatric illnesses and, therefore, the syndrome has a heterogenous presentation. The existing literature states that there is an association between Cotard's delusion with parietal lobe lesions. There is atrophy of the median frontal lobe [11].

In previous case reports, Cotard’s syndrome has been primarily reported in women and older age groups and found to be rarely associated with adolescents [12]. In this clinical study, four cases were male unlike previous reports, and one case belonged to the adolescent age group.

Cotard’s syndrome is attributed to having three developmental stages [13]. The germination stage includes hypochondriasis and constant complaining of abnormal bodily sensations. The blooming stage is characterized by nihilistic delusions. The chronic stage comprises systematizing delusions associated with mood changes [14]. In five of the cases in the series, symptoms occurred gradually and were progressive in nature just like the stages that have been mentioned. In two cases, however, it was seen that fixed and unshakeable belief was present from as early as 15 days.

Berrios and Luque, on the basis of their factor data analysis of 100 cases aged 16-81 years, have reported Cotard’s syndrome having three types [15]. Psychotic depression was characterized by a few nihilistic delusions and melancholia. Cotard type 1 showed pure nihilistic delusion without any depression or other disorders. Cotard type 2 showed a varied presentation comprising depression, anxiety, and third-person auditory hallucination. Previous case reports have mostly found cases belonging to the psychotic depression subtype [16]. Two cases in our series fit into the first subtype of psychotic depression, four cases in the Cotard type 1 subtype, and one case in the Cotard type 2 subtype. This finding is in contrast to other previous findings, which have been reported.

Depression has been reported as the most common diagnosis of Cotard’s syndrome. According to phenomenology, denial of the existence of body organs is reported to be the most common. It is also associated with other forms of nihilistic delusions like denial of mind, intellect, universe, self, and pregnancy. Five cases in this study negated the existence of body organs like the liver, brain, and kidneys. Two cases have negated the existence of the world [16]. Similar to previous reports, both female patients in our study have negated the existence of their self and the soul.

Among the seven cases, six cases believed the non-existence of body organs had led them to self-starvation and nutritional deficiencies. This can be attributed to their nihilistic delusion, which is similar to the cases reported before [8].

One study has reported Cotard’s syndrome in the presence of severe mental retardation in a patient [17]. One case in our study also had a diagnosis of schizophrenia with moderate mental retardation. Studies have also reported the presence of catatonia in Cotard’s syndrome, which was seen later as the symptoms progressed than the nihilistic delusions [18]. However, the cases we have presented in this series were not accompanied by catatonia.

In previous studies, Electroconvulsive Therapy has been the most useful treatment for Cotard’s syndrome when other treatments comprising pharmacotherapy and non-pharmacological treatments have failed [19]. There are studies where patients have benefitted from monotherapy of an antidepressant or an antipsychotic alone or in combination of both [20]. In this series of cases, three patients had improvement in symptoms with a second-generation antipsychotic alone. Two patients have responded to monotherapy with risperidone 6 mg and one patient has responded to Olanzapine 20 mg in our study. One patient has improved on a combination of Pimozide 10 mg and Escitalopram 10 mg. Three cases who have failed to respond to pharmacotherapy alone were treated with Electroconvulsive Therapy and these patients have shown improvement after four to eight sessions.

The study had its limitations. The sample size was small, but we believe it has contributed to the existing literature, as the syndrome is rare. We have not monitored the patients over time; therefore, the lack of follow-up is another limitation of our study.

## Conclusions

Cotard’s syndrome is a rare psychiatric syndrome that is characterized by nihilistic delusions and denial of one’s own existence or the existence of the world. The case series presents a unique finding of a higher proportion of male patients and an adolescent case, as compared to previous reports. The stages of Cotard’s syndrome were found to be present in our cases, with gradual progression in most cases, while some cases had fixed beliefs early on. The study also highlights the different subtypes of Cotard’s syndrome and its associated symptoms, which provides a better understanding of the condition. The most common diagnosis in our cases was depression, and the denial of the existence of body parts was the most prevalent phenomenon. Nihilistic delusions in our cases led to self-starvation and deficiencies. Treatment options for Cotard’s syndrome include pharmacotherapy, electroconvulsive therapy, or a combination of both, with various agents showing effectiveness in improving symptoms. The study provides valuable insights into the diagnosis and treatment of Cotard’s syndrome, which may help in the early recognition and management of this rare condition.
